# Hypoxic microenvironment determines the phenotypic plasticity and spatial distribution of cancer‐associated fibroblasts

**DOI:** 10.1002/ctm2.1438

**Published:** 2023-10-14

**Authors:** Jae‐Il Choi, Eun Jeong Cho, Min Jae Yang, Hyun‐Jin Noh, So Hyun Park, Seokhwi Kim, You‐Sun Kim, Chang Ohk Sung, Dakeun Lee

**Affiliations:** ^1^ Department of Pathology Ajou University School of Medicine Suwon South Korea; ^2^ Department of Biomedical Sciences Ajou University Graduate School of Medicine Suwon South Korea; ^3^ Department of Medical Science Asan Medical Institute of Convergence Science and Technology University of Ulsan College of Medicine Asan Medical Center Seoul Republic of Korea; ^4^ Department of Pathology Asan Medical Center University of Ulsan College of Medicine Seoul Republic of Korea; ^5^ Department of Internal Medicine Ajou University School of Medicine Suwon South Korea; ^6^ Department of Biochemistry and Molecular Biology Ajou University School of Medicine Suwon South Korea

Dear Editor,

Recent single‐cell RNA‐sequencing (scRNA‐seq) techniques have corroborated a previously unappreciated cancer‐associated fibroblast (CAF) heterogeneity in multiple cancer types.^1^ Among several CAF subtypes, myofibroblastic CAFs (myCAFs) and inflammatory CAFs (iCAFs) are consistently observed across various cancer types and constitute major CAF populations.^2^ To date, it has been regarded that certain CAF subtypes are shaped in response to specific cancer‐driven signals and CAFs can transform from one state to another.^3^, ^4^ However, the mechanisms governing the phenotypic shift from myCAFs to iCAFs (or vice versa) remain largely unknown.

To uncover the hidden mechanism responsible for CAF plasticity, first, we compared the differentially expressed transcription factors (TFs) in CAFs with normal tissue fibroblasts (NFs) using seven large‐scale scRNA‐seq datasets (Figure 1A and Figure S1A). We found that only HIF1A (hypoxia‐inducible factor 1‐alpha) and PRRX1 were commonly activated in CAFs (Figure 1B‐C). Our recent discovery of PRRX1 as a master TF that determines myCAF led us to think that HIF1A could be a key factor in establishing iCAF phenotype.^5^ Higher HIF1A expression was common in CAFs than in NFs (Figure 1D and Figure S1B). Trajectory analysis indicated the progressive differentiation of NFs to CAFs, and HIF1A expression gradually elevated with increasing pseudotime (Figure 1E and Figure S1C‐D). The proportion of HIF1A‐expressing CAFs was greatly increased compared to that of NFs in each organ, but not all CAFs expressed HIF1A (Figure 1F), suggesting the heterogeneous nature of CAFs.

**FIGURE 1:**
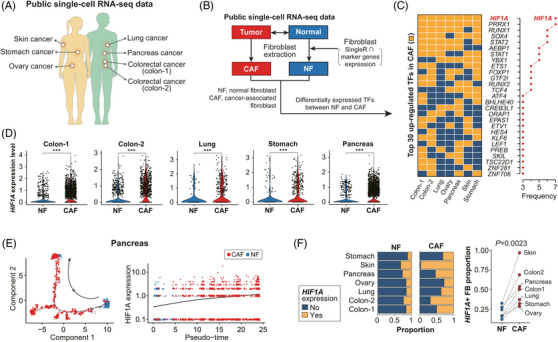
*HIF1A* is a key transcription factor defining CAFs. (A) The seven public scRNA‐seq datasets used in this study. (B) Extraction of fibroblasts of normal and cancer tissue, respectively, from the seven scRNA‐seq data using singleR and fibroblast marker genes. (C) Up‐regulated TFs in CAFs compared with those in NFs across seven datasets. Only *HIF1A* and *PRRX1* were commonly upregulated TFs in all analyzed datasets. (D) Normalized *HIF1A* expression in NFs and CAFs in each cancer type (Wilcoxon rank sum test). (E) The pseudotime progression from NFs to CAFs accompanies increasing *HIF1A* expression. (Left: Points indicate Pearson's correlation coefficient. Error bars indicate the 95% confidence interval for the correlation coefficient. Right: log (*p* value) for Pearson's correlation. Redline: *p* = .05). (F) The proportion of *HIF1A* expressing (*HIF1A*(+)) fibroblasts among various NF and CAF groups. The proportion of *HIF1A*(+) fibroblasts was greatly increased in each CAF group compared to its normal counterpart (Wilcoxon rank sum test). CAF, cancer‐associated fibroblast; NF, normal tissue fibroblast. scRNA‐seq, single‐cell RNA‐sequencing.

From scRNA‐seq data, we found two distinct CAF clusters: one is myCAF while the other is iCAF (Figure 2A). As HIF1A is a master transcriptional regulator of the cellular response to hypoxia, we explored the hypoxia pathway signatures in these clusters. Gene set enrichment analysis (GSEA) demonstrated hypoxia pathway enrichment in iCAFs from pancreatic cancer (Figure 2B), as well as in iCAFs from multiple cancer types (Figure 2C and Figure S2A‐B). Subsequent gene set variation analysis (GSVA) highlighted the striking contrast between the iCAF and myCAF signatures (Figure 2D). Representatively, iCAFs were enriched in pathways including TNFα signaling, hypoxia, apoptosis, and inflammatory response, as previously indicated.^2^ Through extensive literature review, we found that most of these pathways were related to hypoxia, mechanistically (Figure S2C).

**FIGURE 2:**
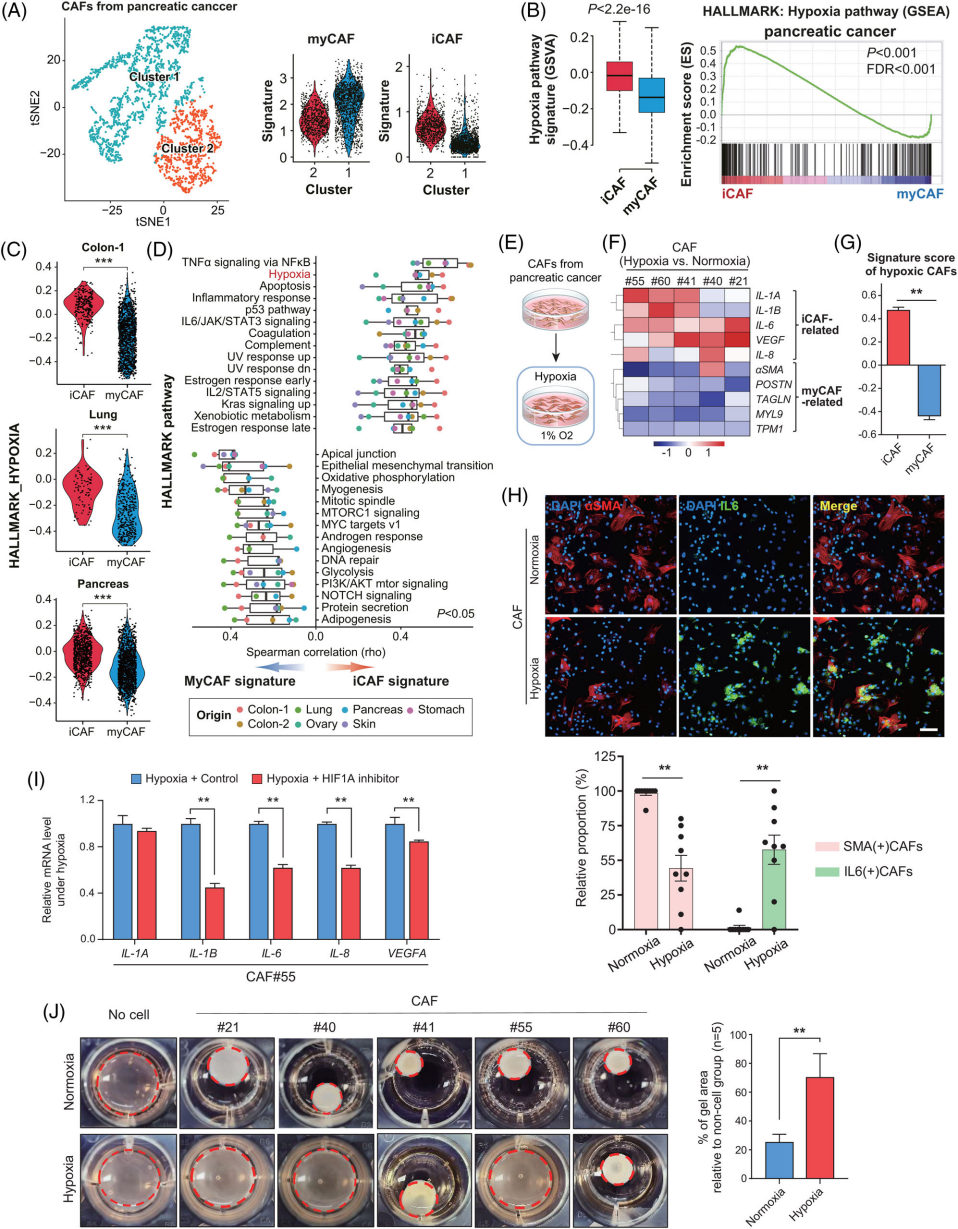
iCAF are enriched with the hypoxia pathway, and CAFs lose myCAF features but gain iCAF features under hypoxia in vitro. (A) Two distinctive subgroups of CAFs from pancreatic cancer are identified in tSNE plot (Left). Cluster 1 shows myCAF signature while cluster 2 shows iCAF signature (Right). (B) Hypoxia signaling pathway analyzed using GSEA is significantly up‐regulated in iCAFs than in myCAFs. (C) iCAFs are associated with upregulated hypoxia signaling pathway (GSVA score for HALLMARK hypoxia gene set) in most cancer types (Wilcoxon rank sum test, ***p < .001). (D) Commonly enriched signaling pathways in iCAF and myCAF across multiple cancer types (Spearman correlation between each CAF subtype signature and HALLMARK gene sets GSVA score). Top 15 signaling pathways for each CAF subtype are shown (points indicating the origin of the dataset). The presented pathways are all statistically significant (Spearman correlation *p* value < .05). (E) Primary CAFs derived from pancreatic cancer are cultured under either normoxia (O_2_ 21%) or hypoxia (O_2_ 1%). (F) Heatmap showing the iCAF‐ and myCAF‐related genes in CAFs cultured under hypoxia compared with those under normoxia is depicted using qRT‐PCR data (*n* = 5). Experiments were done with at least three technical replicates per biological replicates. mRNA level is shown relative to *GAPDH*. (G) iCAF and myCAF signature scores of CAFs cultured under hypoxia. The scores were calculated using the single‐sample GSEA method of GSVA package. (H) (Upper) Representative immunofluorescence figures for αSMA (red), IL‐6 (green) and DAPI (blue) in CAFs cultured under normoxia or hypoxia (upper). Scale bar, 100 μm. (Lower) Quantification of the relative proportions of αSMA(+) or IL‐6(+) CAFs either in normoxia or hypoxia (Two‐way ANOVA with post hoc Bonferroni test: **p < .05). (I) mRNA level of *IL‐1A*, *IL‐1B*, *IL‐6*, *IL‐8* and *VEGFA* were analyzed in CAF#55 treated with HIF1A inhibitor KC7F2 (10 μM) under hypoxic conditions for 72 h. (Bonferroni's multiple comparisons test: **p < .05) (J) Gel contraction assay using CAFs cultured under normoxia or hypoxia for 72 h. Data are presented as the mean ± SEM; *n* = 5 independent experiments (two‐tailed Mann‐Whitney test: **p < .05). CAF, cancer‐associated fibroblast; iCAF, inflammatory CAF; myCAF, myofibroblastic CAF; GSEA, gene set enrichment analysis; GSVA, gene set variation analysis; NF, normal tissue fibroblast; TF, transcription factor.

Next, we determined whether hypoxia shapes iCAFs in vitro. Using five lines of pancreatic CAFs cultured under normoxia or hypoxia (Figure 2E and Figure S3A), we measured the expression of iCAF‐ or myCAF‐related genes (Table S1). In general, the level of cytokines was elevated whereas myCAF‐related genes were commonly repressed in hypoxic CAFs (Figure 2F and Figure S3B). Thus, hypoxic CAFs had significantly higher iCAF‐associated but lower myCAF‐associated GSEA scores (Figure 2G). Immunofluorescence (IF) study revealed that CAFs cultured under normoxia exhibited myCAF features expressing high αSMA but no cytoplastic IL6. Whereas, in hypoxic CAF, the expression of αSMA was markedly reduced, but the expression of IL6 was remarkably increased (Figure 2H). This phenotypic alteration was dependent on HIF1A, as HIF1A inhibitor (KC7F2) reversed the increased expression of iCAF‐related genes under hypoxia (Figure 2I and Figure S3C). Furthermore, myCAF expresses high levels of contractile cytoskeletons resulting in extracellular matrix (ECM) remodeling,^5^ and we observed significant gel contraction in CAFs cultured under normoxia. In contrast, hypoxic CAFs lost the ECM‐contracting capability (Figure 2J). Collectively, these observations indicate that CAFs lose myCAF features but gain iCAF features under hypoxia.

Then, we performed scRNA‐seq using two CAF lines cultured under normoxia or hypoxia (Figure 3A), which demonstrated the separation of hypoxic CAF clusters (Figure 3B) with enrichment of hypoxia pathway (Figure 3C). For further characterization, we performed GSVA on those CAFs, separately (Figure 3D and Figure S4‐5). This almost perfectly mirrored the up‐ or down‐regulated signaling pathways identified in iCAFs of human tissue (Figure 3E). Significantly higher iCAF but lower myCAF signature scores were also noted in hypoxic CAFs (Figure 3F).

**FIGURE 3:**
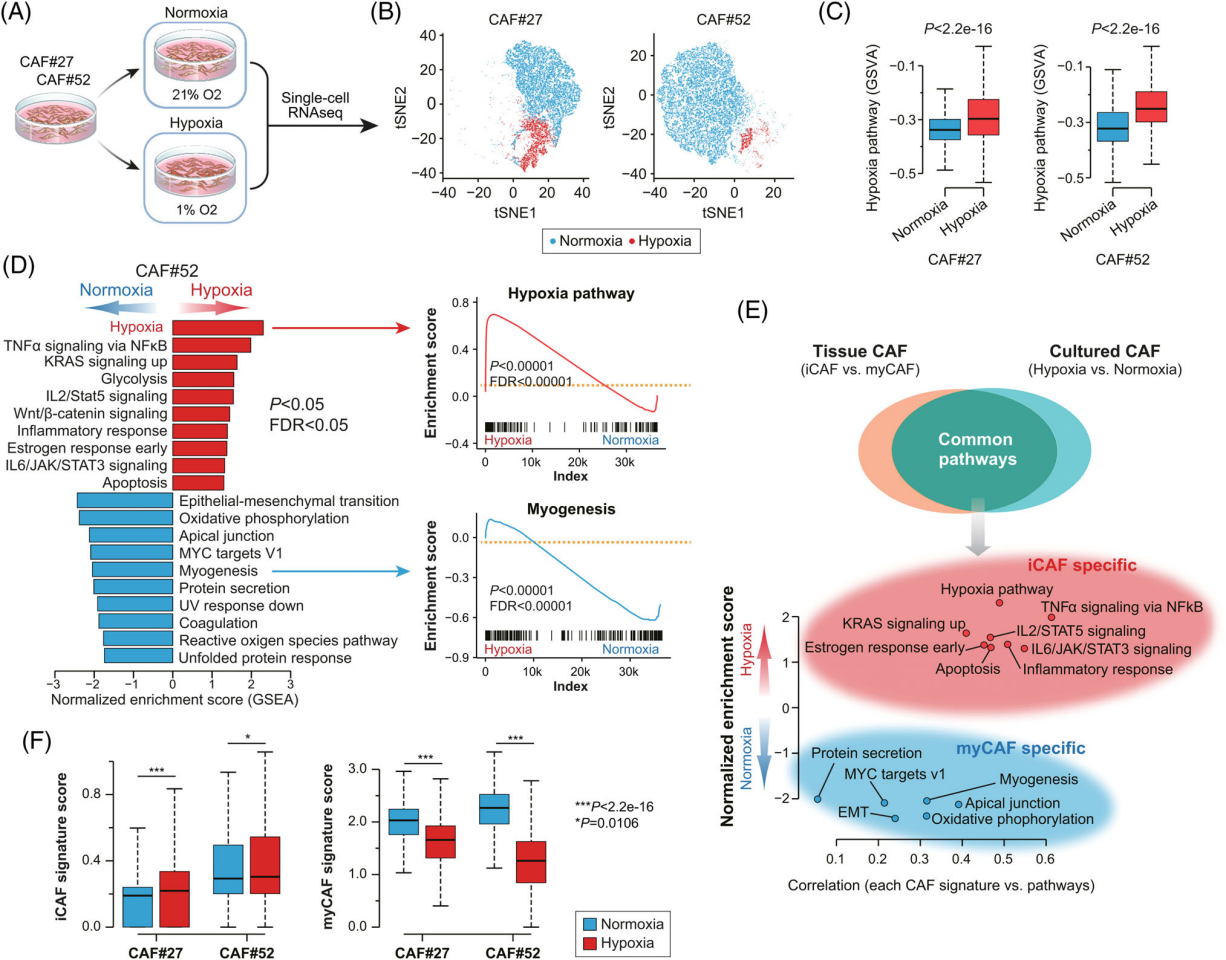
Hypoxic CAFs perfectly mirror iCAFs found in tissue. (A) CAFs obtained from two patients with pancreatic cancer (CAF#27 and CAF#52) were cultured under normoxia (O_2_ 21%) or hypoxia (O_2_ 1%) for three days, then single‐cell RNA‐seq was done using these samples. (B) CAFs cultured under hypoxia are presented as a distinctive subgroup. (C) Hypoxia signaling pathway is enriched in hypoxic CAFs (Wilcoxon rank sum test). (D) Significantly up‐ or downregulated signaling pathways identified in hypoxic CAFs analyzed by GSVA (left) and GSEA (right). (E) Significant overlaps between iCAFs of tissue and CAFs cultured under hypoxia. (F) iCAF and myCAF signature scores in CAF culture under normoxia or hypoxia. CAF, cancer‐associated fibroblast; myCAF, myofibroblastic CAF; iCAF, inflammatory CAF; GSVA, gene set variation analysis; GSEA, gene set enrichment analysis.

For human tissue validation, we examined whether iCAFs preferentially reside in hypoxic regions. To this end, we performed IF on pancreatic cancer tissue and defined iCAFs as IL8‐αSMA double‐positive spindle‐shaped cells. Since the relative αSMA expression in iCAFs is low but still well detected in tissue, we used αSMA to detect both CAF subtypes. As hypoxia typically arises in solid tumours at a distance of approximately 100 μm from a functional blood vessel (Figure S6),^6^ we counted the number of iCAFs in 50‐μm incremental regions from the vessel, with the aid of StrataQuest software (TissueGnostics). As depicted in Figure 4A, iCAFs were scarce in the vicinity of a vessel whereas they were frequently identified in regions distant from the vessel. In the region of interest #3 (ROI#3), the number of iCAFs gradually increased according to the distance from the vessel (Figure 4B), and the same trends were noted in other ROIs (Figure 4C). Finally, we confirmed that the number of iCAFs was significantly increased in distant regions in comparison to the region ≤100 μm from the vessel (Figure 4D), indicating that iCAFs are mainly shaped by the hypoxic TME (Figure 4E).

**FIGURE 4:**
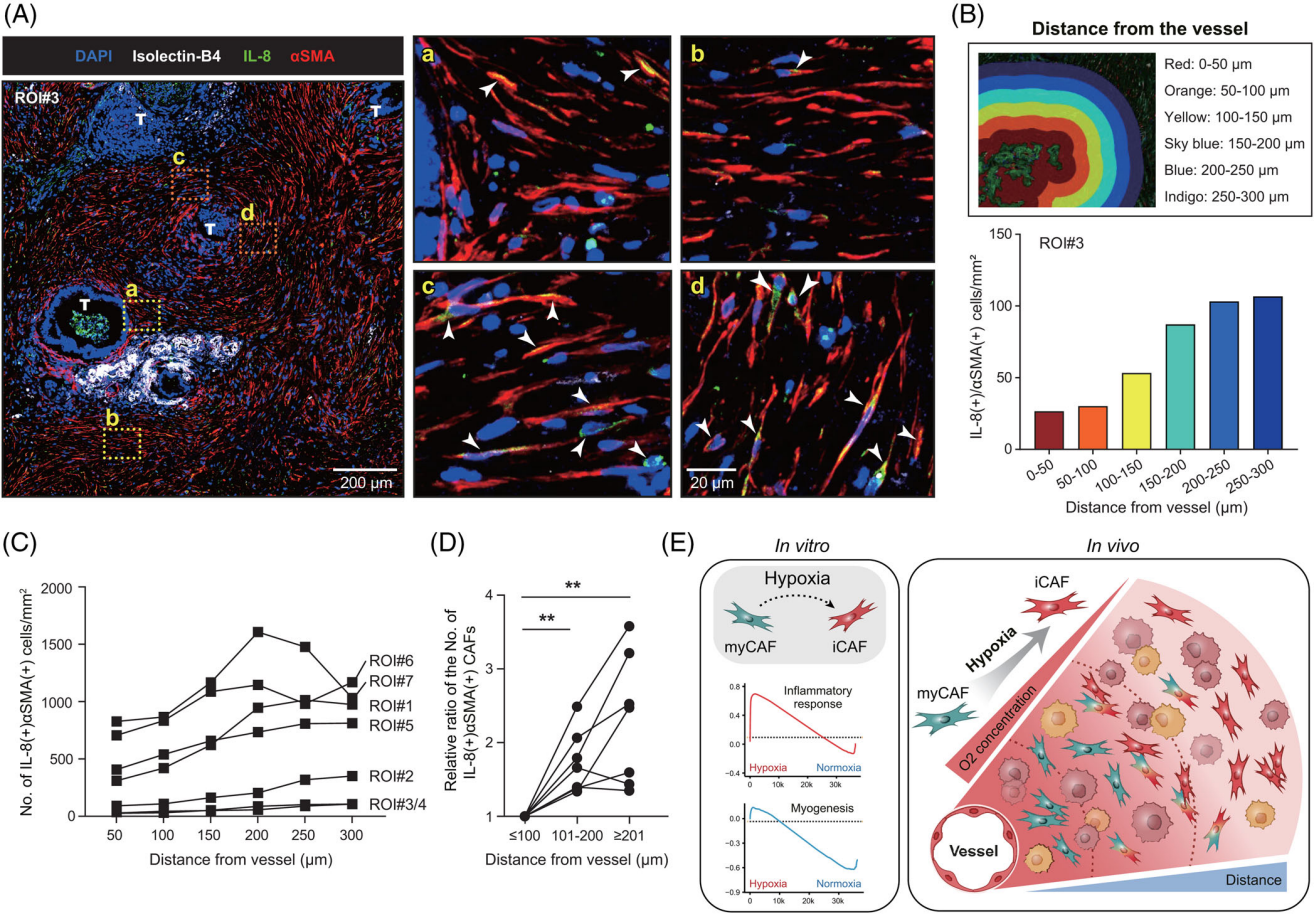
Spatial analysis of cancer tissue reveals the location of iCAFs in a hypoxic region. (A) The representative images of immunofluorescent staining for BS1 Isolectin‐B4 (white), IL‐8 (green), αSMA (red) and DAPI (blue) in human pancreatic cancer tissue. The image was scanned using TissueFAXS. (Left) A representative (ROI#3) low‐magnification image. T indicates tumour. Yellow boxes (“a” and “b”) represent regions near a functional vessel, while orange boxes (“c” and “d”) represent regions away from the vessel. Scale bar, 200 μm. (Right) High‐magnification images for the boxed area of the left image. Arrows indicate spindle cells with yellow cytoplasmic staining (IL‐8 and αSMA double‐positive) CAFs, which were defined as iCAFs in this study. Scale bar, 20 μm. (B) The representative analytic image depicting 50‐μm incremental regions from the vessel (upper) and the barplot showing the number of iCAFs (lower). (C) The number of IL8‐αSMA double‐positive CAFs according to the distance from the vessel across seven different ROIs. (D) The relative ratio of the number of IL8‐αSMA double‐positive CAFs in each indicated region compared to that in the region ≤100 μm from the vessel (two‐tailed Mann–Whitney test: **p < .05). (E) The illustrative summary of this study. CAF, cancer‐associated fibroblast; iCAF, inflammatory CAF; ROI, region of interest; αSMA, alpha‐smooth muscle actin.

To date, it has been regarded that cancer‐secreted IL1 is a key factor in generating iCAF.^4^ However, this specific ligand‐receptor interaction cannot explain the presence of iCAFs in IL1‐negative tumours and the various characteristic signaling pathways of iCAFs. Rather, these are easily explained by ‘hypoxia’ because IL1 can be transcriptionally up‐regulated in hypoxic cells^7^ and HIF1A is a master regulator in establishing iCAF signature as we demonstrated. Moreover, our concept supports the previously suggested premise that iCAFs and myCAFs can reverse from one cell state to another,^3^ as hypoxic regions may dynamically change within tumours according to tumour growth and angiogenesis. In line with our discovery, it was quite recently demonstrated that hypoxia promotes iCAF phenotype in a HIF1α‐dependent manner in a mouse model.^8^ Our results provide additional evidence that this proof of concept is also valid in various human cancers including pancreatic cancer.

In conclusion, our comprehensive analyses indicate that the hypoxic TME is the main driver of the transformation of CAFs into an inflammatory phenotype. Our discovery provides novel insights into CAF biology and strategies for CAF‐directed therapies.

## CONFLICT OF INTEREST STATEMENT

The authors declare that they have no competing interests.

## Supporting information

Supporting Information

Supporting Information

Supporting Information

## Data Availability

The scRNA‐seq data that support the findings of this study are deposited in the Gene Expression Omnibus (GEO) with accession No. GSE223858.
