# Supplementary material for: Hypoxic microenvironment determines the phenotypic plasticity and spatial distribution of cancer‐associated fibroblasts
Source: Clin Transl Med. 2023 Oct 14;13(10):e1438. doi: 10.1002/ctm2.1438 (PMC10576443; doi:10.1002/ctm2.1438)
Supplement: Supplementary file 1 — Supporting Information [file CTM2-13-e1438-s001.docx]

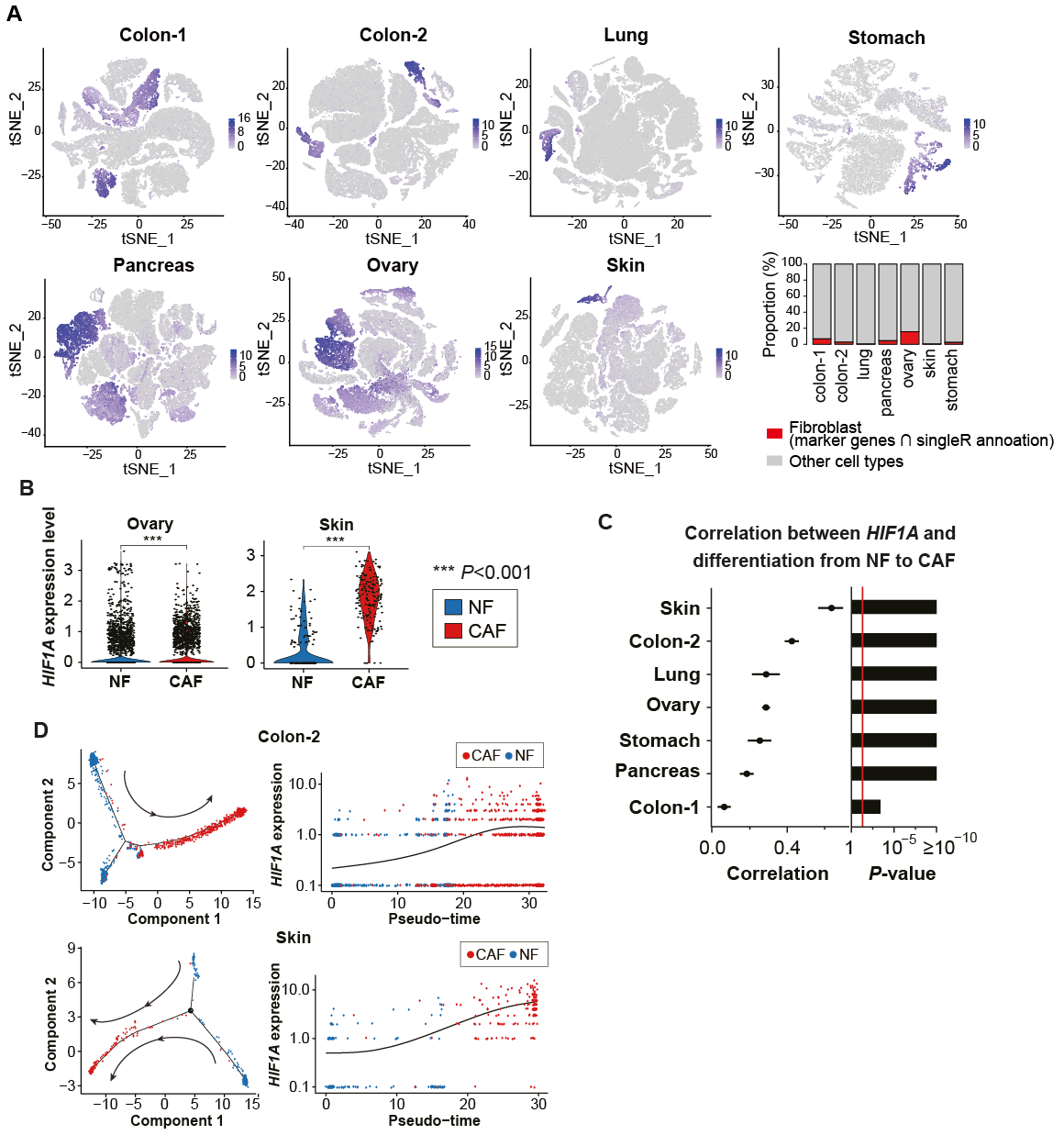


**Figure S1. A.** tSNEplots of single cell RNA-seq obtained from total seven datasets of six organs. Expression (sum) of fibroblast marker genes including *BGN*, *COL1A1*, and *DCN* are predominant in specific clusters, which indicates fibroblast groups. Barplots hows the proportion of fibroblasts defined in this study for each cancer type. **B.** Normalized *HIF1A* expression in NFs and CAFs (Wilcoxon rank sum test). **C.** The pseudotime progression from NFs to CAFs accompanies increasing HIF1A expression. (Left: Points indicate Pearson’s correlation coefficient. Error bars indicate the 95% confidence interval for the correlation coefficient. Right: log (*p* value) for Pearson’s correlation. Redline: *p*= 0.05). **D.** Trajectory (left) and pseudotime (right) analyses between NFs and CAFs in colorectal and skin squamous cell carcinoma. NF, normal fibroblast; CAF, cancer associated fibroblast.


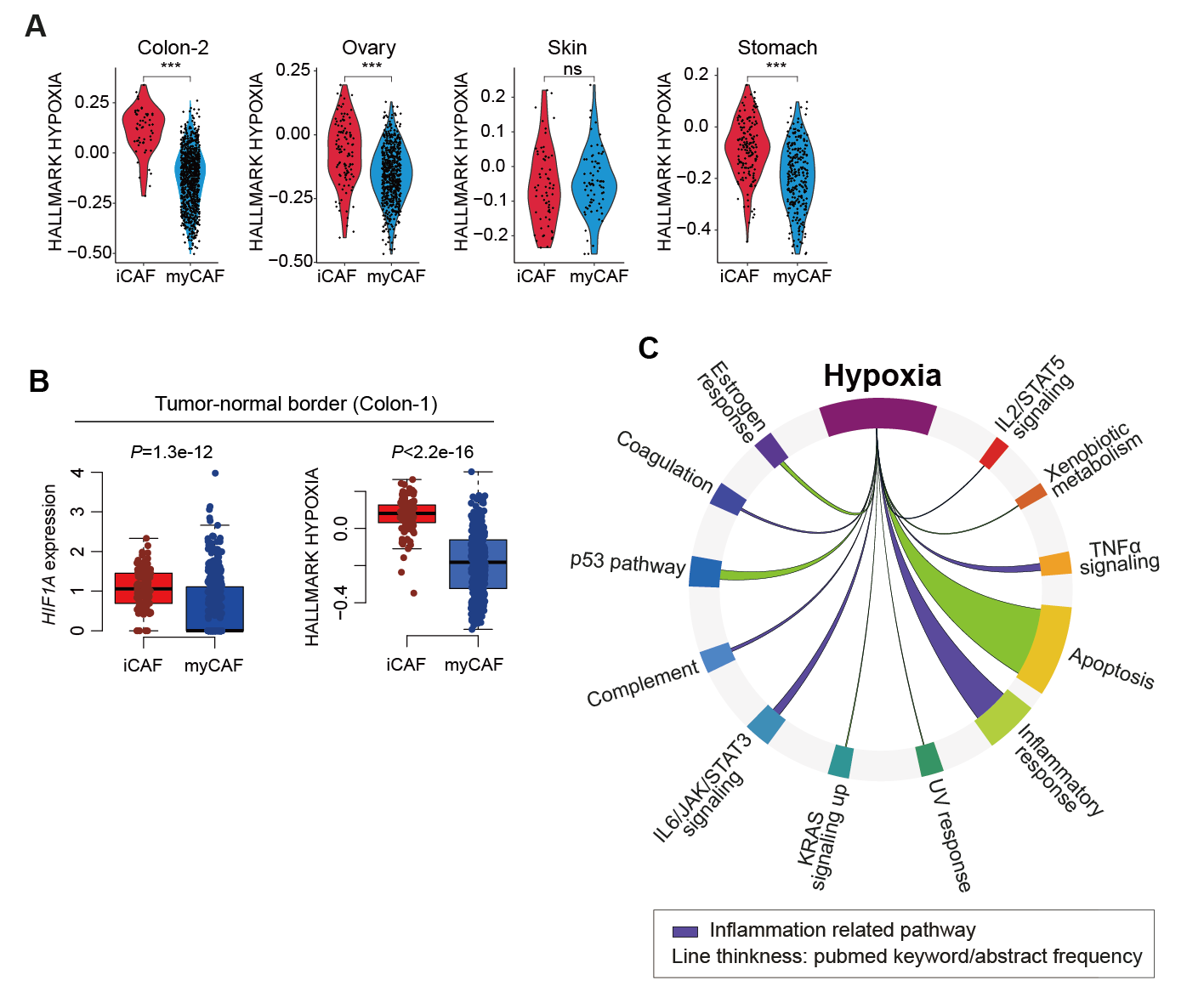


**Figure S2. A.** iCAFs are associated with upregulated hypoxia signaling pathway (GSVA score for HALLMARK hypoxia gene set) in other cancer types, except skin cancer (Wilcoxon rank sum test, ****p*< 0.001, ns > 0.05). **B.** *HIF1A* and hypoxia signaling pathway were also up-regulated in iCAFs compared to myCAFs at the tumor-normal boundary area from Colon-1 dataset (Wilcoxon rank sum test). **C.** A circus plot demonstrates the association between hypoxia and other diverse enriched pathways found in iCAFs. These data were derived from literature review using PubMed. In addition to its association with apoptosis or inflammation-related pathways, such as TNFα signaling and the IL6/JAK/STAT3 pathway, hypoxia triggers the activation of procoagulant pathways or induces complement dysregulation. iCAF, inflammatory cancer-associated fibroblast; myCAF, myofibroblastic cancer-associated fibroblast.


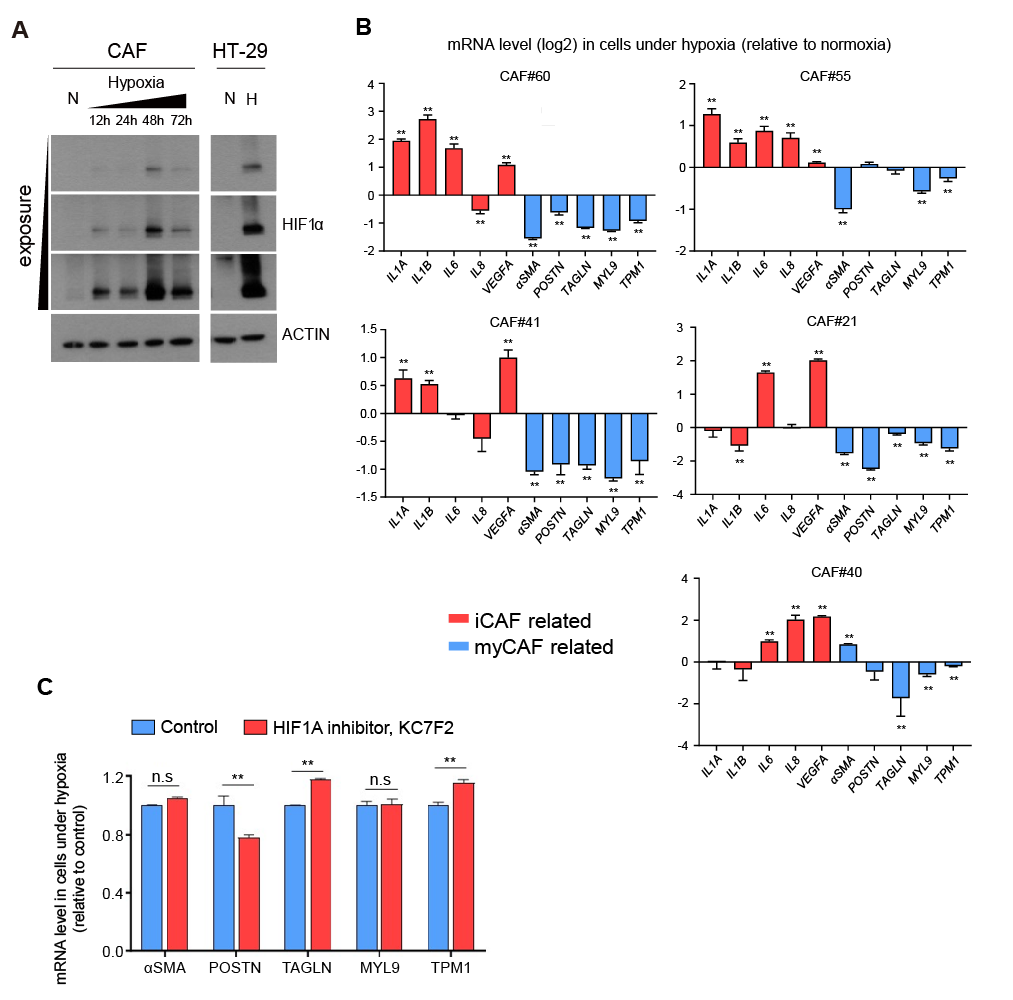
­

**Figure S3. A.** Western blot analyses using cells (CAF#21) cultured under either normoxia (21% O2) or hypoxia (1% O2). HT-29 (colon cancer) cell was used as a positive control. For HT-29, hypoxia was induced for 16 h. N, normoxia; H, hypoxia; HIF1α, hypoxia inducible factor-1α. **B**. qRT-PCR analyses for iCAF-and myCAF-marker genes in cells cultured under either normoxia (21% O2) and hypoxia (1% O2). **C.** mRNA levels of *αSMA, POSTN, TAGLN, MYL9,* and *TPM1* were analyzed in CAF#55 treated with HIF1A inhibitor KC7F2 (10 μM) under hypoxia for 72 h (Bonferroni’s multiple comparis­ons test: ***p* < 0.05). n.s., not significant.


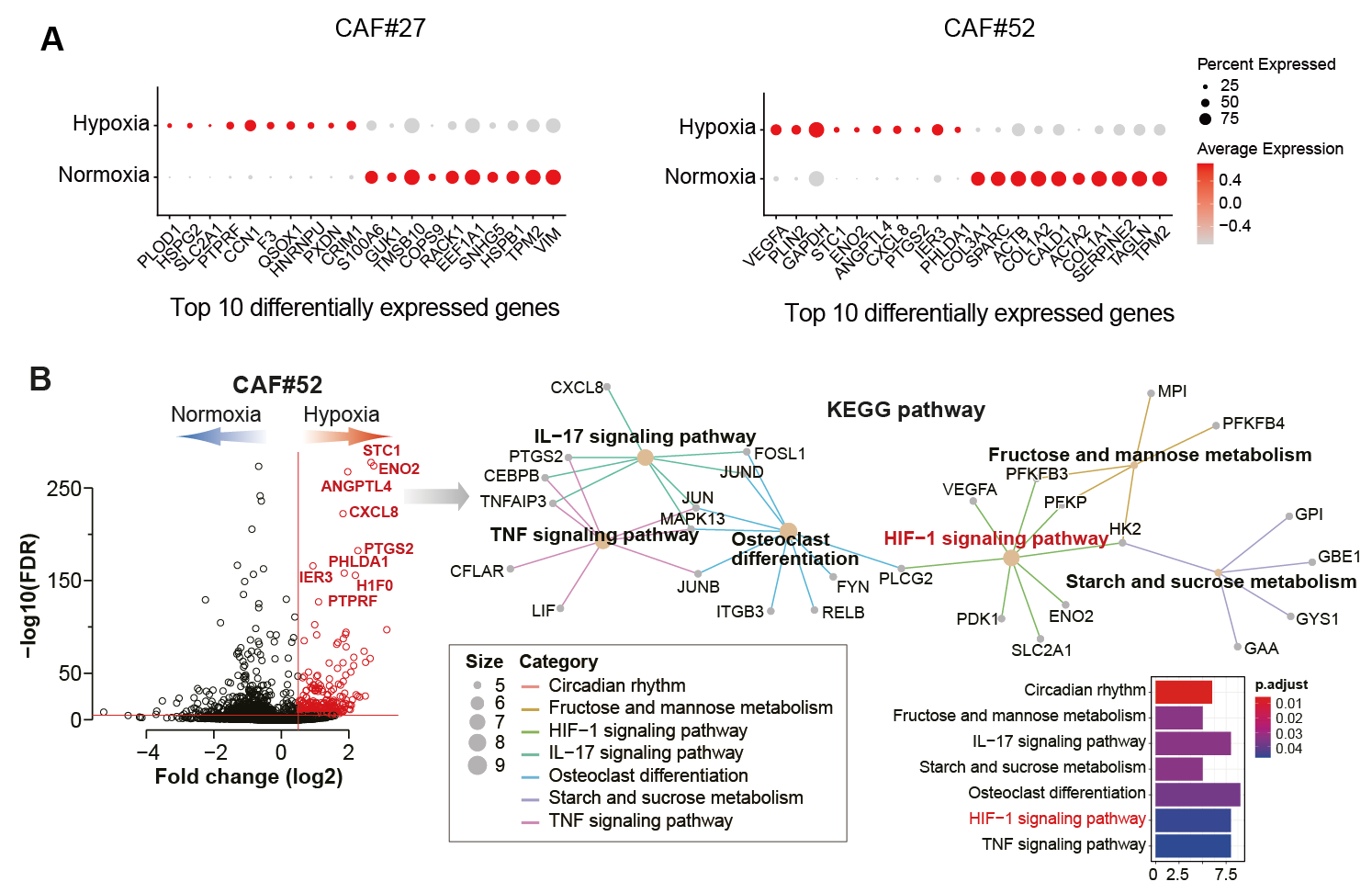


**Figure S4. A.** Single cell RNA-seq analysis using CAFs cultured under either hypoxia (1% O2) or normoxia(21% O2) reveals top upregulated genes (including VEGFA and CXCL8) and top down-regulated genes (including vimentin and αSMA) in hypoxic condition (Wilcoxon rank sum test). **B.**The volcano plot revealed the upregulation of hypoxia-related genes in hypoxic CAFs, including prostaglandin-endoperoxide synthase 2 (*PTGS2*), TNFα induced protein 3 (*TNFAIP3*), FOS like 1 (*FOSL1*), Jun proto-oncogene (*JUN*), leukemia inhibitory factor (*LIF*), integrin subunit beta 3 (*ITGB3*), *VEGFA*, and pyruvate dehydrogenase kinase 1 (*PDK1*). Subsequent gene ontology (GO) analysis demonstrated the enrichment of inflammation-related pathways, such as IL17 or TNF signaling, metabolic pathways, and the HIF1 signaling pathway in hypoxic CAFs.


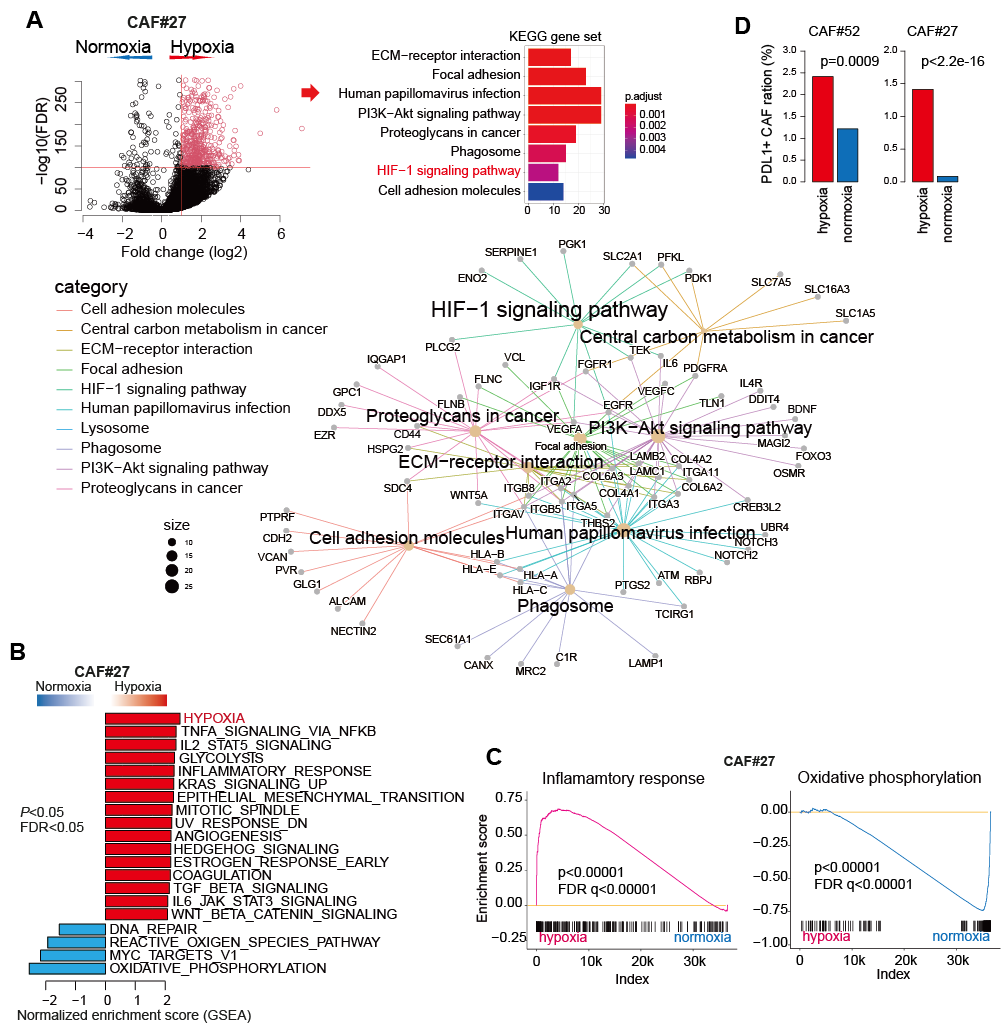


**Figure S5. A.** Gene set analysis using up-regulated genes (Wilcoxon-rank sum test) in CAFs under hypoxia (upper) and the network analysis of the enriched signaling pathways. **B.** Significantly different signaling pathways identified in CAFs under hypoxia in comparison to normoxia using GSVA with Hallmark gene sets. **C.** The representative results of GSEA in CAFs under hypoxia. **D.** PD-L1 positive CAFs are significantly increased in hypoxia condition compared to normoxia (Fisher exact test).


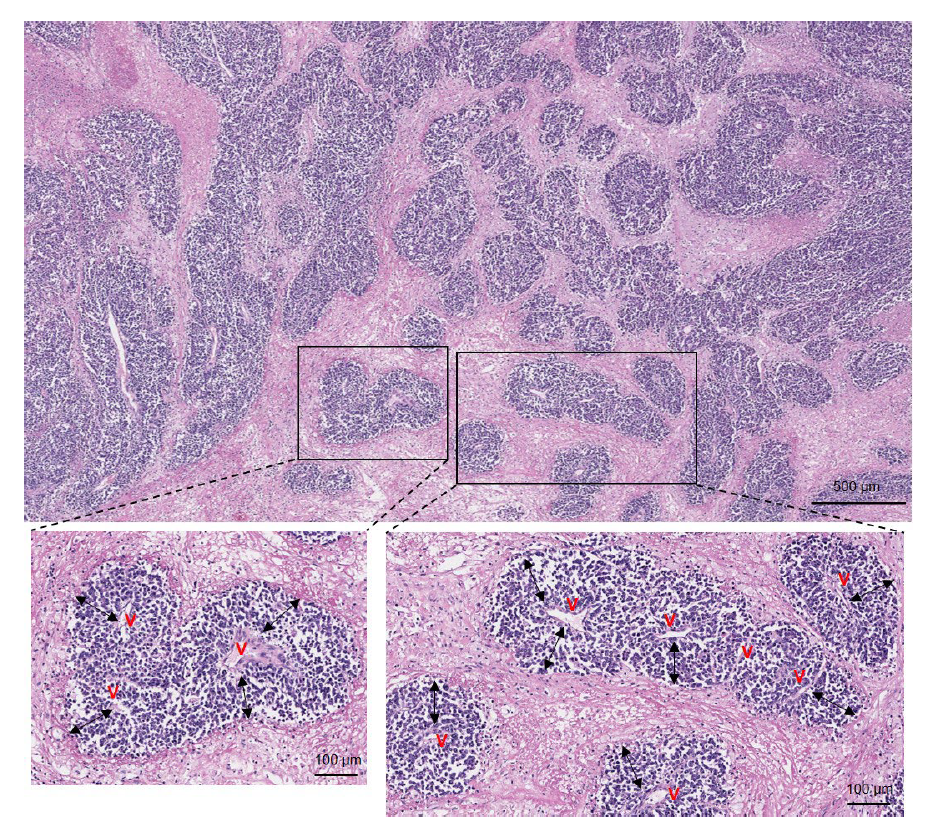


**Figure S6.** Representative photomicrographic images of a case of stromal-lacking neuroendocrine carcinoma of colon. In the low power image, only cancer cells adjacent to blood vessels are viable, whereas the rest underwent ischemic necrosis (pink-colored area). In high power images, viable tumor cells are located up to about 100 μm from the blood vessel. V (red) indicates vessel. Arrows measure 100 μm.


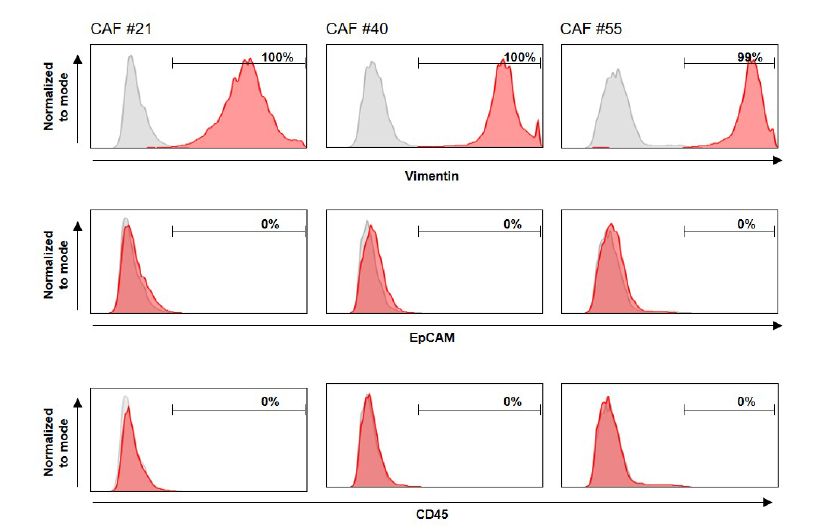


**Figure S7.** Fluorescence-activated cell sorting (FACS) plot showing vimentin (mesenchymal), EpCAM(epithelial), or CD45 (hematopoietic) expression levels on three representative patient-derived CAFs.
