# Supplementary material for: Hypoxic microenvironment determines the phenotypic plasticity and spatial distribution of cancer‐associated fibroblasts
Source: Clin Transl Med. 2023 Oct 14;13(10):e1438. doi: 10.1002/ctm2.1438 (PMC10576443; doi:10.1002/ctm2.1438)
Supplement: Supplementary file 2 — Supporting Information [file CTM2-13-e1438-s002.docx]

**Table S1.** Primer sequences for qRT-PCR.

| **Primer** | **Sequence** |
| --- | --- |
| ***IL-1A*** | Forward 5’-TGCATGGATCAATCTGTGTCTCT-3 |
|  | Reverse 5’-TCAACCGTCTCTTCTTCAGAACC-3 |
| ***IL-1B*** | Forward 5’-AGTGGCAATGAGGATGACTTGTT-3 |
|  | Reverse 5’-GTAGTGGTGGTCGGAGATTCG-3 |
| ***IL-6*** | Forward 5’-CACCTCTTCAGAACGAATTGACA-3 |
|  | Reverse 5’-CCATCTTTGGAAGGTTCAGGTTG-3 |
| ***IL-8*** | Forward 5’-ATACTCCAAACCTTTCCACCCC-3 |
|  | Reverse 5’-CCAGACAGAGCTCTCTTCCATC-3 |
| ***VEGFA*** | Forward 5’-TCAGTTCGAGGAAAGGGAAAGG-3 |
|  | Reverse 5’-GAGGCTCCAGGGCATTAGAC-3 |
| ***αSMA*** | Forward 5’-GGAATGGGACAAAAAGACAGCTAC-3 |
|  | Reverse 5’-GTACTTCAGGGTCAGGATTCCTCTT-3 |
| ***POSTN*** | Forward 5’-CATTGAAGGTGGTGATGGTCATTT-3 |
|  | Reverse 5’-CGACCTTCCCTTAATCGTCTTCT-3 |
| ***TAGLN*** | Forward 5’-AAGTGCAGTCCAAAATCGAGAAG-3 |
|  | Reverse 5’-CCACACTGCACTATGATCCACT-3 |
| ***MYL9*** | Forward 5’-CCACATCCAATGTCTTCGCAAT-3 |
|  | Reverse 5’-GTATTCGTCTGTGGGGTTCTTC-3 |
| ***TPM1*** | Forward 5’-AGATCCAACTGAAAGAGGCCAAG-3 |
|  | Reverse 5’-CAATTCTTCTTCAAGCTCGGCAC-3 |
| ***GAPDH*** | Forward 5’-TTGTCAAGCTCATTTCCTGGTATG-3 |
|  | Reverse 5’-TCTCTCTTCCTCTTGTGCTCTTG-3 |
