# Supplementary material for: Hypoxic microenvironment determines the phenotypic plasticity and spatial distribution of cancer‐associated fibroblasts
Source: Clin Transl Med. 2023 Oct 14;13(10):e1438. doi: 10.1002/ctm2.1438 (PMC10576443; doi:10.1002/ctm2.1438)
Supplement: Supplementary file 3 — Supporting Information [file CTM2-13-e1438-s003.docx]

**Supplementary methods**

**Isolation and culture of CAFs from pancreatic cancer**

CAFs were isolated from endoscopic ultrasound (EUS)-guided biopsy samples from patients with pancreatic cancer and maintained in Dulbecco’s modified Eagle’s medium (HyClone Laboratories) supplemented with 10 FBS, 1% penicillin, and streptomycin. The detailed methods for CAF isolation are described in our recent study [1]. We used CAFs within six passages to avoid potential senescence-associated phenotypic changes.

**Flow cytometry**

To rule out the possibility of epithelial cell and leukocyte contamination, the phenotype of the isolated CAFs was confirmed by positive staining for vimentin, but negative staining for EPCAM and CD45 using flow cytometry (**Figure S7**). CAFs were fixed and permeabilized using the intracellular staining fixation buffer (Biolegend, California, USA) and permeabilization wash buffer (Biolegend) according to the manufacturer’s instructions. After blocking the human Fc receptor (Miltenyi Biotec, North Rhine-Westphalia, Germany), cells were stained with antibodies for Vimentin-Alexa 488 (Biolegend), EpCAM-PE (Biolegend), and CD45-PE (Biolegend). The flow cytometry analysis was performed using FACS Canto2 (BD Biosciences, New Jersey, USA), and data was analyzed using FlowJo (BD Biosciences).

**Single-cell RNA-seq sample preparation**

For single-cell RNA-seq analysis, primary CAFs were incubated in normoxia (21% O_2_) or hypoxia (0.1% O_2_) for 72 h. Hypoxia was achieved by incubating the cells in a hypoxia chamber containing 0.1% O_2_ and 5% CO_2_ and the remainder was N_2_. The chamber was then incubated at 37 °C.

**Single-cell RNA-seq library preparation and sequencing**

The 10x Genomics Chromium platform was used to capture and barcode the cells to generate single-cell Gel Beads-in-Emulsion (GEMs), according to the manufacturer’s protocol. Briefly, along with the reverse transcription master mix, cell suspensions were loaded onto 10x Genomics Single Cell 30 Chips. During this step, the cells were partitioned into GEMs, along with gel beads coated with oligonucleotides. These oligonucleotides enable mRNA capture inside the droplets by 30 bp oligo-dT after cell lysis and provide barcodes to index cells (16 bp) and transcripts (12 bp unique molecular identifiers (UMI)). Following reverse transcription, cDNAs with both barcodes were amplified, and a library was constructed using the Single Cell 3´ Reagent Kit (v3.1 chemistry) for each sample. The resulting libraries were sequenced on an Illumina NovaSeq 6000 System in 2 × 150 bp paired-end mode.

**Sample demultiplexing, barcode processing, and UMI counting**

We performed sample demultiplexing, barcode processing, and UMI counting using the official 10x Genomics pipeline Cell Ranger (v6.1.1) (https://support.10xgenomics.com). Briefly, raw base call files generated by Illumina sequencers were demultiplexed into reads in the FASTQ format using bcl2fastq developed by Illumina (https://github.com/brwnj/bcl2fastq). The raw reads were trimmed from the 3´ end to obtain the recommended number of cycles for read pairs (Read 1: 28 bp; Read 2: 90 bp). The reads from each library were then processed separately using the ‘cellranger count’ pipeline to generate a gene-barcode matrix for each library. During this step, reads were aligned to the human reference genome (GRCh38). Cell barcodes and UMIs associated with the aligned reads were subjected to correction and filtering, and the count matrix data were pre-processed using the Seurat R package (v4.1.1) [2]. UMIs of less than 401 expressed genes, larger than 6,000 and smaller than 200, and more than 20% of reads mapped to mitochondrial RNA were filtered. For visualization, we performed principal component analysis with 2,000 highly variable genes for initial dimensionality reduction and tSNE to reduce PCA dimensions into 2D space.

**Identifying fibroblasts from processed gene expression data of scRNAseq**

After generating tSNE using Seurat, we used two different methods independently and extracted fibroblasts. First, we used known fibroblast marker genes (*COL1A1*, *BGN*, and *DCN*) [3, 4] and the FeaturePlot function on the tSNE data to identify fibroblasts. Second, we used the SingleR package (v1.8.1) [5] to annotate the cell types, including fibroblasts. From these two independent outputs, we specified overlapping cells as fibroblasts in each sample and used them for subsequent analysis.

**Identification of CAF subtypes**

Signature genes from a previous study [6] were used to identify myCAFs (*ACTA2*, *TAGL*, *MMP11*, *MYL9*, *HOPX*, *POSTN*, *TPM1*, and *TPM2*) and iCAFs (*IL6*, *PDGFRA*, *CXCL12*, *CFD*, *DPT*, *LMNA*, *AGTR1*, *HAS1*, *CXCL1*, *CXCL2*, *CCL2*, and *IL8*) for our defined fibroblasts. Each signature was measured using the mean gene expression.

**Differentially expressed transcriptional factors**

We compared the gene expression of CAFs with that of NFs in each dataset. We selected genes that were expressed in at least 10% of all cells in each dataset. Differentially expressed genes were computed using the Wilcoxon test, and genes were filtered based on an FDR < 0.05. The transcription factor list was downloaded from the CIS-BP (<http://cisbp.ccbr.utoronto.ca>, Build 2.00) [7]. Differentially expressed genes upregulated in CAFs were filtered using the TFs list.

**Trajectory analysis in scRNA-seq**

We constructed single-cell trajectories in datasets combining NFs and CAFs. Monocle2 (v2.22.0) [8] was used to calculate the pseudotime for each cell. The trajectory was constructed according to the unsupervised analysis method by selecting genes (mean_expression ≥ 0.1 and num_cells_expressed > 10). Each dataset’s dimensionality was reduced using the ‘DDRTree’ algorithm and ordered by the pseudotime. The state that contained more NFs was considered the root_state. To show the trajectory in each dataset and *HIF1A* expression according to the pseudotime ‘plot_cell_trajectory’ and ‘plot_genes_in_pseudotime’ functions were used.

**Gene set enrichment and pathway analysis**

GSEA (v4.1.0, Mac App) [9] or the GSEAplot v0.1.0 R package [10] were used to identify which gene sets were enriched in the two cell subtypes. We used hallmark gene sets from the Molecular Signatures Database (MsigDB v7.2) or KEGG database for pathway analysis. To assess the gene set enrichment scores of individual cells, GSVA scores were also calculated from the normalized gene expression data using GSVA v1.24.2 [11]. For the identified differentially expressed genes, DisGeNet functional enrichment analysis was conducted using clusterProfiler v4.4.4 [12] and the enrichplot v1.16.1 R package. We applied the enrichDGN function using filtered differentially expressed gene lists for the enrichment analysis of disease-gene associations and visualized enriched terms using the cnetplot function based on the default p-value < 0.05. The strength of the association of the hallmark pathways was visualized using the R package ‘circlize’ v0.4.15 [13] based on the frequency of simultaneous appearance in PubMed (https://pubmed.ncbi.nlm.nih.gov/) abstracts and keywords.

**scRNA-seq datasets from public databases**

Seven public scRNA-seq datasets were used for this study, and two colorectal cancer datasets [3, 14] were obtained from the Gene Expression Omnibus (GEO) with GSE132465 and GSE144735. Ovary cancer and lung cancer datasets were obtained from the ArrayExpress database under accession numbers E-MTAB-8107, E-MTAB-6149, and E-MTAB-6653 [3]. The gastric cancer dataset was obtained from <https://dna-discovery.stanford.edu> [15]. The skin squamous cell carcinoma dataset was obtained from GSE144236 [16]. The pancreatic cancer dataset was obtained from the Genome Sequence Archive (GSA) CRA001160 under the project PRJCA001063 [17]. We normalized the scRNA-seq count matrix data with the NormalizeData function using Seurat R package v4.1.1 [2]. UMIs of less than 401 expressed genes, larger than 6,000 and smaller than 200, and more than 25% of reads mapped to mitochondrial RNA were filtered.

**Quantitative RT-PCR**

Total RNA was isolated using the RNeasy Plus kit (QIAGEN, Venlo, Netherlands), and cDNA was synthesized using the GoScript reverse transcription system (Promega, Wisconsin, USA) according to the manufacturer’s instructions. qRT-PCR was performed with SyBR (Promega) using a Thermal Cycler Dice Real Time System III (TaKaRa Bio, Shiga, Japan). The relative mRNA levels of the target genes were normalized to *GAPDH* levels and analyzed using the comparative Ct method (^ΔΔ^Ct). The heatmap for ^ΔΔ^Ct was visualized using the pheatmap package (v.1.0.12), and single-sample GSEA (ssGESA) [18] scores for iCAF (*IL1A*, *IL1B*, *IL6*, *IL8*, and *VEGF*) and myCAF (*aSMA*, *POSTN*, *TAGLN*, *MYL9*, and *TPM1*) were calculated using the GSVA package (v1.42.0) [11]. The primer sequences used in this study are listed in **Supplementary Table S1**.

**HIF1A inhibition**

CAFs were cultured under hypoxic conditions for 72 h following pre-treatment with HIF1A inhibitor KC7F2 (10 µM; Selleckchem, Texas, USA) for 4 h. mRNA level were determined using qRT-PCR.

**Western blotting**

Cell lysis was carried out in M2 buffer containing 20 mM Tris-HCl (pH 7.6), 0.5% NP-40, 250 mM NaCl, 2 mM EDTA, 3 mM EGTA, 2 mM DTT, 0.5 mM PMSF, 20 mM β-glycerol phosphate, 1 mM sodium vanadate, and 1 μg/ml leupeptin. Equal amounts of cell extracts were resolved by SDS-PAGE and analyzed by immunoblotting. The antibodies used for immunoblotting were as follows: anti-HIF1α (610958, 1:1000; BD Biosciences, New Jersey, USA) and anti-actin (47778, 1:5000; Santa Cruz Biotechnology, California, USA).

**Gel contraction assay**

The collagen gel contraction assay was performed using a Contraction Assay Kit (Cell Biolabs, San Diego, USA) according to the manufacturer’s instructions. Briefly, CAF cells were incubated for 48 h under hypoxic conditions in an atmosphere of 0.1% O2 and 5% CO2 in the Modular Incubator Chamber (Billups-Rothenberg, San Diego, USA) or normoxic conditions. Then, the cells were suspended in collagen gel working solution, and cell-collagen mixtures were loaded into a 24-well cell contraction plate. After incubation for an additional 24 h under both hypoxic and normoxic conditions, collagen gel size changes were measured and quantified using ImageJ software.

**Immunofluorescence staining**

Immunofluorescence (IF) analysis was performed as previously described [19] with minor modifications. CAFs were incubated under hypoxic or normoxic conditions for three days, and after fixation and blocking, cells were stained using antibodies specific for the following: anti-αSMA (Abcam, Cambridge, UK), anti-IL6 (Thermo Fisher Scientific, Massachusetts, USA), Alexa 488 anti-rat (Thermo Fisher Scientific), and Alexa 594 anti-mouse (Thermo Fisher Scientific). Imaging was performed using a confocal microscope (Nikon, Tokyo, Japan).

Paraffin-embedded sections were deparaffinized as previously described [20]. After heat-induced epitope retrieval using citrate buffer, sections were blocked with goat serum and BSA for 60 min at room temperature. The sections were then incubated with FITC-conjugated isolectin B4 (Sigma-Aldrich, St. Louis, MO, USA), anti-αSMA (Abcam), and anti-IL8 (Abcam). After washing, the sections were stained with the appropriate secondary antibodies for 1 h. The use of patient’s sample was approved by the Institutional Review Board of Ajou University Hospital (AJIRB-BMR-SMP-20-222).

**Imaging analysis**

Images of IF-stained pancreatic cancer samples were obtained using the TissueFAXS PLUS Cytometer platform (TissueGnostics, Vienna, Austria). After recognizing FITC-positive functional vessels with a diameter between 50 and 200 μm, we detected spindle-shaped cells with cytoplasmic αSMA expression, drew an outline of these cells, and then identified CAFs. We defined αSMA-IL8 double-positive spindle cells as iCAFs. To distinguish the iCAF populations, we set appropriate cutoff values using cytometric scatter plots, where each cutoff was verified by backward connection. Finally, the cells were quantified according to their distance from the vessel using StrataQuest software (TissueGnostics).

**Statistical analysis**

Statistical analyses were performed using GraphPad Prism 9 or R version 4.2.1. The experimental data are presented as mean ± SEM, and differences were compared using the two-tailed Mann-Whitney U test. Two-way ANOVA were performed using Bonferroni’s multiple comparisons test. Correlation analysis of the continuous variables was performed using Spearman’s correlation analysis. The Wilcoxon rank-sum test (non-parametric) was used to evaluate the significance of the differences in continuous variables between the two groups.

**References**

1. Kim S, Woo KJ, Yang CM, Park SH, Hwang JC, Yoo BM, et al. Simultaneous establishment of pancreatic cancer organoid and cancer-associated fibroblast using a single-pass endoscopic ultrasound-guided fine needle biopsy specimen. Dig Endosc. 2023. <https://doi.org/10.1111/den.14648>.

2. Hao Y, Hao S, Andersen-Nissen E, Mauck WM, 3rd, Zheng S, Butler A, et al. Integrated analysis of multimodal single-cell data. Cell. 2021;184(13):3573-87.e29. <https://doi.org/10.1016/j.cell.2021.04.048>.

3. Qian J, Olbrecht S, Boeckx B, Vos H, Laoui D, Etlioglu E, et al. A pan-cancer blueprint of the heterogeneous tumor microenvironment revealed by single-cell profiling. Cell Res. 2020;30(9):745-62. <https://doi.org/10.1038/s41422-020-0355-0>.

4. Chung HC, Cho EJ, Lee H, Kim WK, Oh JH, Kim SH, et al. Integrated single-cell RNA sequencing analyses suggest developmental paths of cancer-associated fibroblasts with gene expression dynamics. Clin Transl Med. 2021;11(7):e487. <https://doi.org/10.1002/ctm2.487>.

5. Aran D, Looney AP, Liu L, Wu E, Fong V, Hsu A, et al. Reference-based analysis of lung single-cell sequencing reveals a transitional profibrotic macrophage. Nat Immunol. 2019;20(2):163-72. <https://doi.org/10.1038/s41590-018-0276-y>.

6. Elyada E, Bolisetty M, Laise P, Flynn WF, Courtois ET, Burkhart RA, et al. Cross-Species Single-Cell Analysis of Pancreatic Ductal Adenocarcinoma Reveals Antigen-Presenting Cancer-Associated Fibroblasts. Cancer Discov. 2019;9(8):1102-23. <https://doi.org/10.1158/2159-8290.Cd-19-0094>.

7. Weirauch MT, Yang A, Albu M, Cote AG, Montenegro-Montero A, Drewe P, et al. Determination and inference of eukaryotic transcription factor sequence specificity. Cell. 2014;158(6):1431-43. <https://doi.org/10.1016/j.cell.2014.08.009>.

8. Qiu X, Mao Q, Tang Y, Wang L, Chawla R, Pliner HA, et al. Reversed graph embedding resolves complex single-cell trajectories. Nature Methods. 2017;14(10):979-82. <https://doi.org/10.1038/nmeth.4402>.

9. Subramanian A, Tamayo P, Mootha VK, Mukherjee S, Ebert BL, Gillette MA, et al. Gene set enrichment analysis: a knowledge-based approach for interpreting genome-wide expression profiles. Proc Natl Acad Sci U S A. 2005;102(43):15545-50. <https://doi.org/10.1073/pnas.0506580102>.

10. Innis SE, Reinaltt K, Civelek M, Anderson WD. GSEAplot: A Package for Customizing Gene Set Enrichment Analysis in R. J Comput Biol. 2021;28(6):629-31. <https://doi.org/10.1089/cmb.2020.0426>.

11. Hanzelmann S, Castelo R, Guinney J. GSVA: gene set variation analysis for microarray and RNA-seq data. BMC Bioinformatics. 2013;14:7. <https://doi.org/10.1186/1471-2105-14-7>.

12. Wu T, Hu E, Xu S, Chen M, Guo P, Dai Z, et al. clusterProfiler 4.0: A universal enrichment tool for interpreting omics data. Innovation (Camb). 2021;2(3):100141. <https://doi.org/10.1016/j.xinn.2021.100141>.

13. Gu Z, Gu L, Eils R, Schlesner M, Brors B. circlize Implements and enhances circular visualization in R. Bioinformatics. 2014;30(19):2811-2. <https://doi.org/10.1093/bioinformatics/btu393>.

14. Lee HO, Hong Y, Etlioglu HE, Cho YB, Pomella V, Van den Bosch B, et al. Lineage-dependent gene expression programs influence the immune landscape of colorectal cancer. Nat Genet. 2020;52(6):594-603. <https://doi.org/10.1038/s41588-020-0636-z>.

15. Sathe A, Grimes SM, Lau BT, Chen J, Suarez C, Huang RJ, et al. Single-Cell Genomic Characterization Reveals the Cellular Reprogramming of the Gastric Tumor Microenvironment. Clin Cancer Res. 2020;26(11):2640-53. <https://doi.org/10.1158/1078-0432.Ccr-19-3231>.

16. Ji AL, Rubin AJ, Thrane K, Jiang S, Reynolds DL, Meyers RM, et al. Multimodal Analysis of Composition and Spatial Architecture in Human Squamous Cell Carcinoma. Cell. 2020;182(2):497-514.e22. <https://doi.org/10.1016/j.cell.2020.05.039>.

17. Peng J, Sun BF, Chen CY, Zhou JY, Chen YS, Chen H, et al. Single-cell RNA-seq highlights intra-tumoral heterogeneity and malignant progression in pancreatic ductal adenocarcinoma. Cell Res. 2019;29(9):725-38. <https://doi.org/10.1038/s41422-019-0195-y>.

18. Barbie DA, Tamayo P, Boehm JS, Kim SY, Moody SE, Dunn IF, et al. Systematic RNA interference reveals that oncogenic KRAS-driven cancers require TBK1. Nature. 2009;462(7269):108-12. <https://doi.org/10.1038/nature08460>.

19. Hong SM, Lee AY, Hwang SM, Ha YJ, Kim MJ, Min S, et al. NAMPT mitigates colitis severity by supporting redox-sensitive activation of phagocytosis in inflammatory macrophages. Redox Biol. 2022;50:102237. <https://doi.org/10.1016/j.redox.2022.102237>.

20. Bae WJ, Kim S, Ahn JM, Han JH, Lee D. Estrogen-responsive cancer-associated fibroblasts promote invasive property of gastric cancer in a paracrine manner via CD147 production. FASEB J. 2022;36(11):e22597. <https://doi.org/10.1096/fj.202200164RR>.
